# Natriuretic peptide type C induces sperm attraction for fertilization in mouse

**DOI:** 10.1038/srep39711

**Published:** 2017-01-05

**Authors:** Nana Kong, Xiaoting Xu, Yu Zhang, Yakun Wang, Xiaoqiong Hao, Yu Zhao, Jie Qiao, Guoliang Xia, Meijia Zhang

**Affiliations:** 1 State Key Laboratory for Agrobiotechnology, College of Biological Sciences, China Agricultural University, Beijing 100193, China; 2 Department of Obstetrics and Gynecology, Reproductive Medical Center, Peking University Third Hospital, Beijing 100191, China

## Abstract

Mammalian spermatozoa undergo selective movement along the isthmus of the oviduct to the ampulla during ovulation, which is a prerequisite for fertilization. The factor(s) that involves in selective spermatozoa movement is still unknown. In this study, we found that the oviductal epithelium in mouse ampulla expressed high levels of natriuretic peptide type C (NPPC) in the presence of ovulated oocyte-cumulus complexes (OCCs). Spermatozoa expressed NPPC receptor natriuretic peptide receptor 2 (NPR2, a guanylyl cyclase) on the midpiece of flagellum. NPPC increased intracellular levels of cGMP and Ca^2+^ of spermatozoa, and induced sperm accumulation in the capillary by attraction. Importantly, spermatozoa from *Npr2* mutant mice were not attracted by NPPC, preventing fertilization *in vivo*. Oocyte-derived paracrine factors promoted the expression of *Nppc* mRNA in the ampulla. Therefore, NPPC secreted by oviductal ampulla attracts spermatozoa towards oocytes, which is essential for fertilization.

Oocytes attract spermatozoa by secreting chemical factors to promote fertilization. In animals with external fertilization, species-specific sperm chemoattractant proteins bind to membrane guanylyl cyclase receptors^1^,^2^, or the receptors associated with guanylyl cyclases^3^ on the sperm flagellum, and stimulate rapid synthesis of cyclic guanosine monophosphate (cGMP)^4^,^5^. Ca^2+^ entering through a K^+^-selective cGMP-gated ion channel^6^,^7^ increases flagellar asymmetry, resulting in chemotactic movement^8^,^9^ along the gradient of chemoattraction^4^,^10^,^11^. Currently, chemotaxis has not been definitively established in mammalian sperm.

In mammals, a considerable fraction of the spermatozoa inseminated rapidly reaches the storage site in the isthmus of the oviduct with reduced motility^12^, but only a few spermatozoa recover their motility and swim from the storage to the fertilization site in the ampulla when ovulation occurs^13^,^14^. Experimental data suggest chemical attraction for spermatozoa near the oocyte in the ampulla, to trigger fertilization^15^. The signal originates in the oocyte microenvironment^16^,^17^, including follicular fluid^18^,^19^,^20^, oviductal fluid^19^ and oocyte-conditioned media^21^, which is most likely conducive to capacitated spermatozoa^22^,^23^ and is correlated with fertilization success^24^.

Amino acid sequence analysis suggests that mouse natriuretic peptides (NPs), including type A (NPPA, also known as ANP), type B (NPPB, also known as BNP) and type C (NPPC, also known as CNP), exhibit features similar to the chemoattractant peptides in marine invertebrates (see Supplementary Fig. S1). Further, NPPA attracts mammalian spermatozoa *in vitro*^25^,^26^, and NPPC increases human sperm motility^27^. However, the chemoattraction mechanism of naturally occurring attractant(s) after ovulation remains unclear. The aim of this study was to investigate the expression pattern of NPPC in mouse oviduct and its cognate receptor natriuretic peptide receptor 2 (NPR2) on spermatozoa, and their role in sperm attraction and fertilization.

## Results

### *Nppc* mRNA expression in mouse ampulla depends on stimulation of ovulated oocyte-cumulus complexes (OCCs)

**Figure 1:**
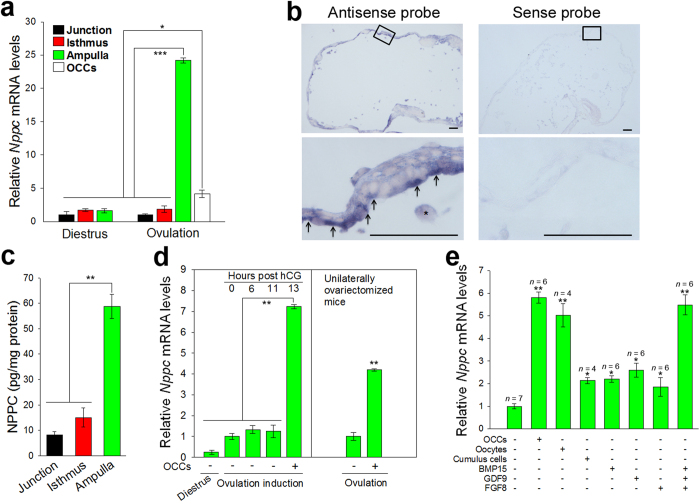
Expression of *Nppc* mRNA by epithelium of oviductal ampulla. (**a**) Comparison of steady-state levels of *Nppc* mRNA in the ampulla, isthmus, uterotubal junction (junction) and ovulated oocyte-cumulus complexes (OCCs) isolated from mice at diestrus or ovulation. The mean value in the uterotubal junction from mice at diestrus was set as 1. Bars indicate the mean ± SEM of three experiments. *P < 0.05; ***P < 0.001. (**b**) *In situ* hybridization showing *Nppc* mRNA expression in epithelium (arrows) of oviductal ampulla. Frozen sections of ampullae were hybridized with DIG-labeled antisense probe detecting *Nppc* mRNA (left panels), and the sense probe detecting control group (right panels). Sense probes yielded only background staining. Asterisks (*) indicate the cumulus cell. Scale bars, 50 μm. (**c**) Comparison of NPPC levels in the ampulla, isthmus, and uterotubal junction of mice at ovulation. Bars indicate the mean ± SEM of three experiments. **P < 0.01. (**d**) Effect of ovulation on *Nppc* expression in ampulla. *Nppc* mRNA levels in the ampullae were increased only in the presence of the ovulated OCCs. In the left panel, the mean value of the ampullae isolated at 0 h post hCG was set as 1. Bars indicate the mean ± SEM of three experiments. **P < 0.01. (**e**) The effects of OCCs, cumulus cells, oocytes and oocyte-derived paracrine factors on *Nppc* expression in ampullae. Ampullae isolated from preovulatory mice (at 11 h post hCG) were cocultured with OCCs, oocytectomized (OOX) cumulus cells, denuded oocytes (oocyte; three oocytes/μL), or oocyte-derived paracrine factors human GDF9 (500 ng/mL), human BMP15 (500 ng/mL) and human FGF8B (FGF8, 100 ng/mL), or the combination of the three proteins for 3 h and levels of *Nppc* mRNA were determined. The mean value in the control (no treatment) group was set as 1. Bars indicate the mean ± SEM; *n*, number of independent replicates. *P < 0.05; **P < 0.01.

In general, species-specific chemoattractant proteins are secreted to attract the spermatozoa during ovulation^4^,^28^. Therefore, the gene expression of natriuretic peptides in mouse oviduct was analyzed using quantitative reverse transcription-polymerase chain reaction (qRT-PCR). *Nppc* mRNA was expressed predominantly in the ampulla of estrous mice, and its levels were dramatically higher than those of *Nppa* and *Nppb* mRNAs (see Supplementary Fig. S2a,b). Induction of ovulation also resulted in high levels of *Nppc* mRNA in the ampulla (Fig. 1a). Expression of *Nppc* mRNA in the ampulla was further determined by *in situ* hybridization. *Nppc* mRNA was expressed predominantly by the oviductal epithelium (Fig. 1b) lining the inside of the ampulla. The protein levels of NPPC were detected with fluorescent enzyme immunoassay in the oviducts of mice following ovulation. The concentration of NPPC in the ampulla (58.8 ± 4.8 pg/mg protein) was significantly higher than in the uterotubal junction and isthmus (Fig. 1c).

During ovulation, the released oocyte-cumulus complexes (OCCs) reside in the ampullae awaiting fertilization^29^. Higher levels of *Nppc* mRNA were detected in the ampullae after ovulation (Fig. 1d). Therefore, we determined the possible role of ovulated OCCs in regulating *Nppc* mRNA levels. Co-culture of ampullae with OCCs dramatically promoted *Nppc* mRNA expression (Fig. 1e). However, OCCs expressed low levels of *Nppc* mRNA (Fig. 1a) as reported previously^30^. Thus, the levels of *Nppc* mRNA in ampulla are regulated by ovulated OCCs. The effects of oocytes and cumulus cells derived from OCCs were further determined. Microsurgical extirpation of oocytes from complexes (OOX cumulus cells) only partially promoted *Nppc* mRNA expression by ampullae *in vitro* (Fig. 1e). However, co-culture of ampullae with denuded oocytes (three oocytes/μL) restored *Nppc* mRNA levels equivalent to those promoted by co-culture with OCCs, suggesting that the levels of *Nppc* mRNA in the ampulla are regulated by oocyte-derived paracrine factors. Growth differentiation factor 9 (GDF9), bone morphogenetic protein 15 (BMP15) and fibroblast growth factor 8B (FGF8) are paracrine growth factors secreted by oocytes^31^. Each of these growth factors only slightly promoted the expression of *Nppc* mRNA by ampulla (Fig. 1e). However, the combination of the three proteins restored the levels of *Nppc* mRNA equivalent to those promoted by co-culture with oocytes or OCCs (Fig. 1e).

### NPR2 is located on the flagellar midpiece of spermatozoa

**Figure 2:**
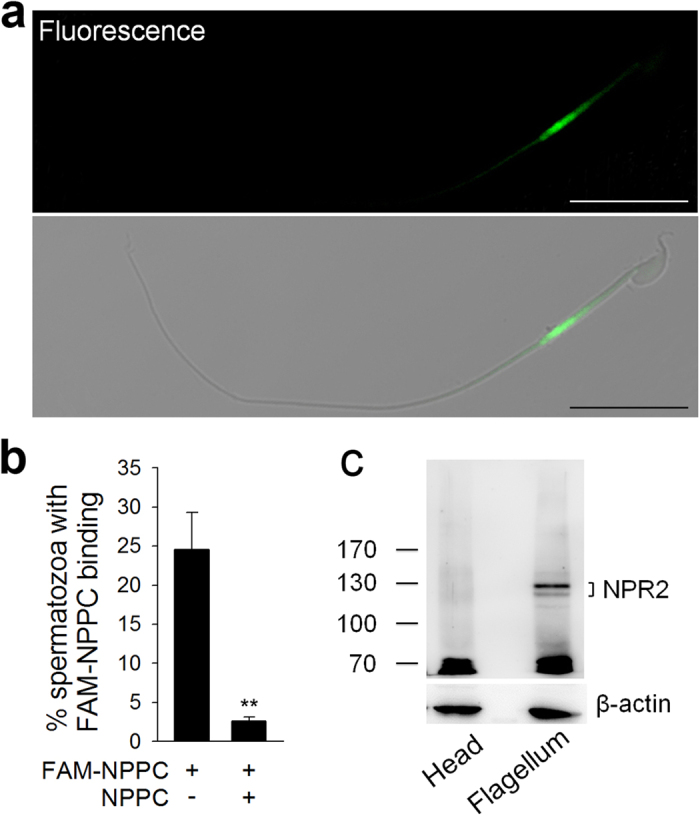
Expression of NPR2 on sperm flagellum. (**a**) Capacitated spermatozoa were incubated with FAM-NPPC (100 nM) for 30 min to ensure fully reaction. The binding of FAM-NPPC to the spermatozoon as visualized by fluorescence (up panel) and merge (down panel) fields. Scale bar, 20 μm. (**b**) Comparison of FAM-NPPC binding in spermatozoa without or with NPPC (1 μM) competition. Bars indicate the mean ± SEM of three experiments. *n* = 500 for each group. **P < 0.01. (**c**) A representative western blot of NPR2 from sperm heads and flagella. NPR2 antibody recognized two immunoreactive bands that were obvious in the fraction of sperm flagella.

Typically, chemoattractants bind to sperm surface receptors^26^,^32^,^33^. NPPC acts locally as an autocrine and paracrine regulator via natriuretic peptide receptor 2 (NPR2), a guanylyl cyclase-coupled receptor^30^,^34^. The levels of NPPC receptor *Npr2* mRNA were detected in spermatozoa (see Supplementary Fig. S3a). To test the location of NPR2 on the sperm, we used a fluorescent-labeled ligand NPPC (FAM-NPPC) to bind with the receptor. This method is widely used for location of chemoattractant receptor on non-mammalian sperm^2^. The green fluorescence, representing FAM-NPPC binding site, was observed on the midpiece of flagellum, especially on the distal midpiece (Fig. 2a, see Supplementary Fig. S3c–e). The positive signal was seen in 23.6 ± 4.4% of capacitated sperm (Fig. 2b). Few spermatozoa (2.6 ± 0.5%) showed fluorescence staining in the samples and competitive binding in the presence of excess unlabeled peptide (1 μM NPPC) (Fig. 2b). Furthermore, few capacitated spermatozoa derived from *Npr2* mutant mice showed FAM-NPPC binding (see Supplementary Fig. S4). All these results indicate that NPR2 was the binding site of FAM-NPPC on mouse sperm. However, the positive staining in fresh spermatozoa was only 2.3 ± 0.3% (see Supplementary Fig. S3f). The endogenous levels of NPR2 were further detected using immunoprecipitation followed by Western blot, which confirmed that NPR2 was expressed predominantly in sperm flagellum (Fig. 2c). Two immunoreactive bands (~130 kDa and ~117 kDa) correspond to NPR2 with different amounts of glycosylation as observed in previous studies of mouse and rat granulosa cells^35^,^36^,^37^.

### NPPC induces sperm accumulation in the capillary

**Figure 3:**
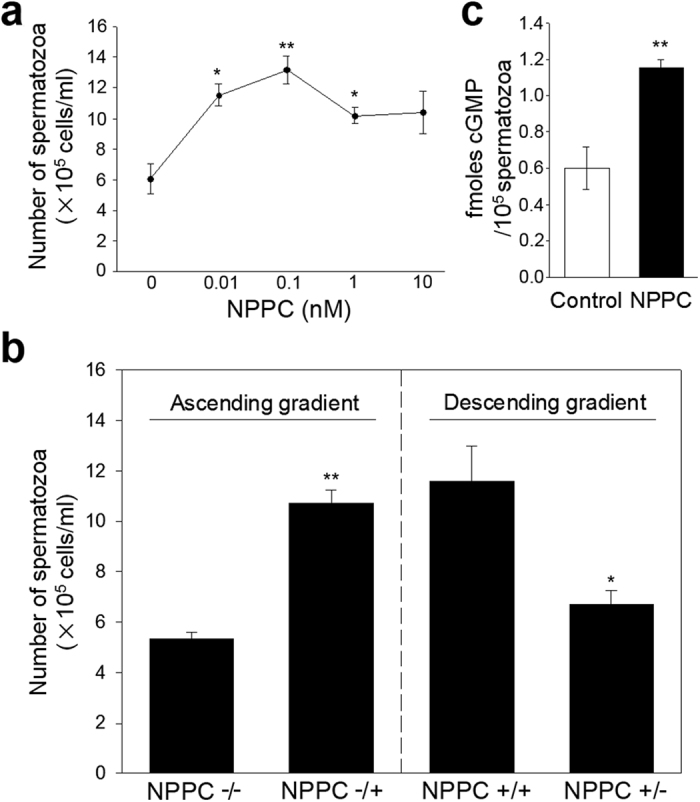
NPPC induces sperm accumulation in the capillary. (**a**) Effect of NPPC on sperm accumulation in the capillaries at different concentrations. After capacitation, spermatozoa were added to a 150 mL droplet at a final concentration of 1–2 × 10^6^ cells/mL, and capillaries were exposed to various doses of NPPC. The number of spermatozoa in the capillaries was counted after incubation for 20 min. Bars indicate the mean ± SEM of three experiments with three repeats per group in each experiment. *P < 0.05; **P < 0.01. (**b**) Effect of NPPC on sperm accumulation in the capillaries with ascending and descending gradients. For the ascending gradient, the droplet had no NPPC, and the capillary was supplemented without (−/−) or with 0.1 nM NPPC (−/+). For the descending gradient, the droplet contained 0.1 nM NPPC, and the capillary was supplemented without (+/−) or with 0.1 nM NPPC (+/+). Bars indicate the mean ± SEM of three experiments with two repeats per group in each experiment. *P < 0.05; **P < 0.01. (**c**) Effect of NPPC on cGMP levels in spermatozoa. Capacitated spermatozoa were incubated without or with 1 nM NPPC for 20 min, and the amounts of cGMP were evaluated using enzyme immunoassay kits. Bars indicate the mean ± SEM of four experiments. **P < 0.01.

The intriguing juxtaposition of the cell types expressing the ligand NPPC and its cognate receptor NPR2 suggests a functional relationship. We found that NPPC enhanced straight-line velocity of the sperm (Table 1). NPPC significantly increased sperm accumulation in the capillaries, and the concentration (to form optimal chemoattractant gradient) is 0.1 nM (Fig. 3a). Furthermore, the number of spermatozoa in the capillaries under descending gradient (Fig. 3b, right panel, NPPC+/− group) was significantly decreased compared with that in the absence of any gradient at all (NPPC+/+ group). Thus, NPPC induces sperm accumulation by attraction, which is most likely attributed to chemokinesis and chemotaxis. The rate of FAM-NPPC binding was further increased in the attracted spermatozoa (see Supplementary Fig. S3f).

### NPPC elevates intracellular cGMP and Ca^2+^ levels in spermatozoa

**Figure 4:**
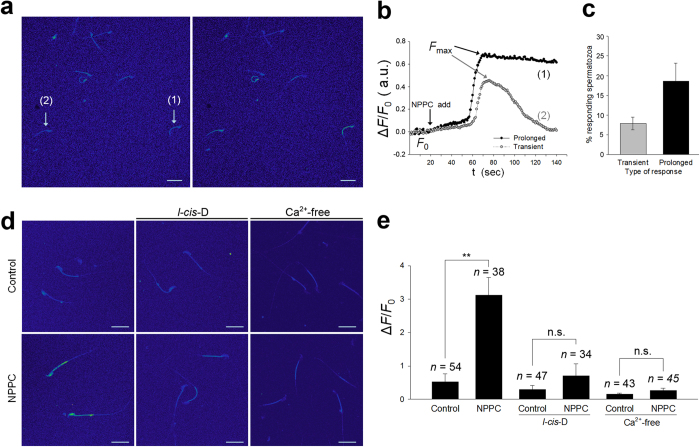
NPPC induces Ca^2+^ influx in mouse spermatozoa. (**a**) Micrographs of mouse spermatozoa before (left panel) and after (right panel) stimulation with 0.1 nM NPPC. Images were presented in a pseudocolor format. Scale bars, 20 μm. (**b**) Representative fluorescence intensity-time relationship showing transient and prolonged responses. The traces showed the maximal fluorescence intensity (*F*_max_) in sperm head and flagellar midpiece after the addition of NPPC (NPPC add) in two spermatozoa (1 and 2, in **a**). F_0_ represents the baseline fluorescence before application of stimulus. (**c**) Percentage of each type of response induced by NPPC. Bars indicate the mean ± SEM of four experiments. *n* = 31 to spermatozoa responding. (**d**) Representative micrographs of spermatozoa without or with 0.1 nM NPPC incubation (left panel) or supplemented with 50 μM *l-cis*-Diltiazem (*l-cis*-D; middle panel) for 1 min, or without or with NPPC incubation for 1 min in Ca^2+^-free medium (right panel). Scale bars, 20 μm. (**e**) Comparison of normalized mean fluorescence intensity response (Δ*F*/*F*_0_) in the sperm head and flagellar midpiece after different treatments. Bars indicate the mean ± SEM of five experiments. *n*, number of spermatozoa for each bar. **P < 0.01.

Stimulation of spermatozoa by chemoattractants increases intracellular cGMP levels, and triggers a rapid and transient elevation of intracellular Ca^2+^, which is essential for attraction^4^,^32^,^38^,^39^,^40^. In the present study, NPPC significantly increased the intracellular levels of cGMP in spermatozoa (Fig. 3c). Next, we examined the effect of NPPC on Ca^2+^ levels in spermatozoa by measuring the changes in fluorescence intensity. The fluorescence intensity was dramatically increased in the head and flagellar midpiece in response to NPPC (Fig. 4a,d). As reported before^41^, NPPC induced a transient response (7.9 ± 1.6%), where Ca^2+^ levels returned to baseline within 140 sec (Fig. 4b,c), and a prolonged response (18.6 ± 4.5%), where Ca^2+^ levels remained high and returned to baseline after 140 sec. NPPC induced Ca^2+^ response in 26.5% of the sperm population, consistent with the FAM-NPPC binding rate in capacitated spermatozoa. In addition, we observed a time lag of 2–3 sec in the response between the head and the flagellar midpiece (see Supplementary Fig. S5), consistent with other studies that a tail to head propagation of a calcium wave is found in response to several agents including 8-Br-cGMP^11^,^41^,^42^,^43^. This finding indicates that Ca^2+^ elevation starts in the flagellar midpiece, and immediately propagates toward the head. The initial site of Ca^2+^ elevation in spermatozoon is consistent with NPR2 distribution.

Ca^2+^ entering via cGMP-sensitive cyclic nucleotide-gate (CNG) channels plays an important role in sperm attraction in marine invertebrates^7^. Subsequently, we used *l-cis-*Diltiazem (*l-cis*-D, a CNG-channel inhibitor) to determine the role of CNG channels in NPPC-induced Ca^2+^ elevation. The addition of 0.1 nM NPPC increased the maximum fluorescence intensity three-fold (Fig. 4d,e), whereas pre-incubation of spermatozoa with 50 μM *l-cis*-D completely inhibited NPPC-induced increase in Ca^2+^ fluorescence intensity (Fig. 4d,e). Treatment with *l-cis*-D also inhibited NPPC-induced sperm accumulation in the capillary and oviductal ampulla (see Supplementary Fig. S6). Furthermore, the removal of Ca^2+^ from the medium completely blocked NPPC-induced increase in Ca^2+^ fluorescence intensity (Fig. 4d,e). Thus, NPPC induces sperm accumulation and Ca^2+^ influx by CNG channels.

### Failure of NPPC-induced attraction and artificial insemination of spermatozoa in *Npr2* ^cn-2J^/*Npr2* ^cn-2J^ mutant mice

**Figure 5:**
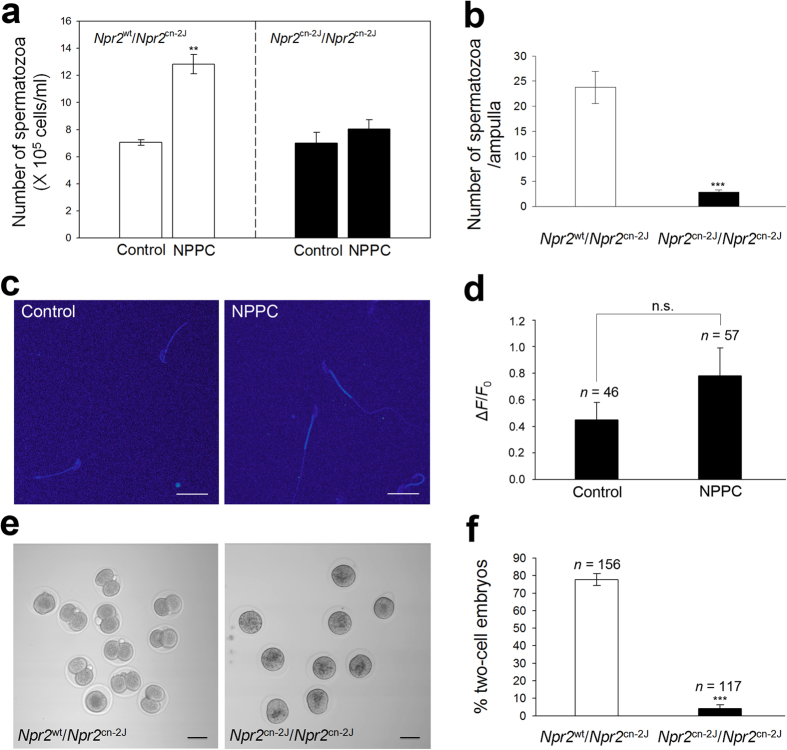
Failure of NPPC-induced attraction and artificial insemination of spermatozoa from *Npr2*^cn-2J^/*Npr2*^cn-2J^ mutant mice. (**a** and **b**) Accumulation of spermatozoa in the capillaries of NPR2 heterozygous and mutant males (**a**) and in the oviductal ampullae (**b**). Bars indicate the mean ± SEM of three experiments, each repeated twice per group. **P < 0.01, ***P < 0.001. (**c**) Representative spermatozoon micrographs of NPR2 mutant mice without or with 0.1 nM NPPC incubation for 1 min. Scale bars, 20 μm. (**d**) Comparison of normalized mean fluorescence intensity response (Δ*F*/*F*_0_) in the sperm head and flagellar midpiece of NPR2 mutant mice after different treatments. Bars indicate the mean ± SEM of four experiments. *n*, number of spermatozoa for each bar. (**e**) Images of two-cell embryos after artificial insemination with spermatozoa from NPR2 heterozygous and mutant mice. Embryos and presumptive zygotes were collected from the oviducts 36 h after artificial insemination. Scale bar, 50 μm. (**f**) Percentages of two-cell embryo using spermatozoa of NPR2 heterozygous and mutant males. Bars indicate mean ± SEM of three experiments, each repeated four times per group. *n*, number of embryos and presumptive zygotes examined. ***P < 0.001.

We hypothesized that the spermatozoa of *Npr2* mutant mice are not attracted to oocytes, if the attraction was mediated via the NPPC/NPR2 pathway. Spermatozoa were collected from adult *Npr2*^cn-2J^/*Npr2*^cn-2J^ homozygous mutant and *Npr2*^wt^/*Npr2*^cn-2J^ heterozygote mice and analyzed. NPPC failed to induce accumulation (Fig. 5a) and Ca^2+^ response (Fig. 5c,d) of spermatozoa from *Npr2* mutant mice. *Npr2* mutant males show normal spermatogenesis^44^, and the spermatozoa exhibited normal motility (see Supplementary Table S1) and the ability for fertilization *in vitro*^45^. However, these mutant males fail to mate naturally due to erectile dysfunction (ED) accompanying a trapped foreskin^44^. Therefore, we investigated their ability for *in vivo* fertilization via artificial insemination (AI). After 4 h, the spermatozoa arriving at the ampullae were counted. The number of spermatozoa from *Npr2*^wt^/*Npr2*^cn-2J^ heterozygous males was 23.8 ± 3.2 in each ampulla. However, few spermatozoa (2.9 ± 0.5) were observed in *Npr2*^cn-2J^/*Npr2*^cn-2J^ mutant males (Fig. 5b). After 1.5 days, the rate of two-cell embryos was 77.7 ± 3.4% with spermatozoa derived from *Npr2* heterozygous males, and was only 5.4 ± 1.7% in *Npr2* mutant males (Fig. 5e,f). Therefore, NPPC/NPR2 plays an essential role in attracting spermatozoa for fertilization.

## Discussion

In the present study, we showed that ovulation stimulated NPPC, which bound to NPR2 on spermatozoa, to increase attraction for oocytes that reside in the ampulla, and ensure normal fertilization. Unlike marine invertebrates, the mammalian spermatozoa are placed inside the female genital tract without interspecific sperm competition, and species specificity for sperm attraction is not observed^46^. Interestingly, the sequence of the chemoattractant peptide NPPC in mammals is highly conserved^47^.

A stable long-lasting attraction is a prerequisite for fertilization. After the LH surge, mammalian OCCs are ovulated into the oviductal ampullae, awaiting fertilization. It is reported that oocyte arrival in the oviduct results in a change in the oviductal gene expression^48^, which plays important roles in gamete transport, fertilization and embryo development. In our study, *Nppc* mRNA levels are dramatically increased locally. *In vitro* studies further showed that oocytes and oocyte-derived paracrine factors stimulated the transcription of *Nppc* in the ampullae, which resulted in high levels of NPPC protein. These results are consistent with previous studies suggesting that oocyte-stimulated SMAD signaling promoted *Nppc* mRNA expression in granulosa cells^49^,^50^. Thus, the timing of the availability of NPPC in oviductal ampulla is programmed to the availability of oocytes at this site. It promoted a stable attractant gradient in the oviduct, which is consistent with previous studies showing that spermatozoa exhibit an attractive response and swim from the storage area to the fertilization site of ampulla during ovulation^13^,^14^. Progesterone has been a candidate as the attractant the mammalian follicular fluid^51^,^52^,^53^. However, sperm attraction is not correlated with progesterone levels in the follicular fluid^18^,^24^. Progesterone induces sperm accumulation probably by inducing hyperactivation^54^. Interestingly, the follicular fluid of pre-ovulatory follicles contains a high concentration of NPPC produced by mural granulosa cells to maintain oocyte meiotic arrest^30^,^36^,^55^. However, NPPC concentration in the follicular fluid dramatically decreases during ovulation^55^,^56^. Furthermore, tiny amounts of follicular fluid are released at the onset of ovulation^22^, which prevent formation of a stable and persistent attractive gradient along the oviduct lumen. Recently, it is reported that coitus induces oviductal flow that clears the oviduct of cellular debris and provides rheotactic guidance for sperm^57^. Anyway, the oviductal flow is beneficial for the establishment of a chemoattractant gradient in the oviduct. On the other hand, myosalpinx contractions play important roles in the formation of sperm assemblage in the isthmus, and in the transport of the assemblage to the middle region of the oviduct^58^. Thus, the range of sperm chemotaxis in the oviduct may be relatively short. In the previous study, the ovulated oocytes without cumulus matrix are unfertilized, leading to severe female infertility^59^. In the present study, cumulus cells slightly expressed *Nppc* mRNA and also stimulated the transcription of *Nppc* mRNA in the ampullae. It needs further study whether cumulus cells also participate in this attractive response.

FAM-NPPC bound to NPR2 at the midpiece of sperm. While few fresh spermatozoa showed NPR2 positive staining (FAM-NPPC binding), the staining was increased in capacitated spermatozoa (see Supplementary Fig. S3f), consistent with previous studies^22^,^26^. The increase in NPR2-positive spermatozoa may be related to unmasking of NPR2^26^, since *Npr2* mRNA levels were not increased by capacitation (see Supplementary Fig. S3b) and the sperm nuclear transcription is in a dormant state^60^. A quarter of spermatozoa showed NPR2-positive staining, probably due to the fact that only a small fraction of spermatozoa is capacitated at a given time^22^. The number of spermatozoa with NPR2-positive staining was further increased after treatment with NPPC, suggesting that only the spermatozoa with functional NPR2 are attracted by NPPC. This is consistent with recent studies that only a few spermatozoa migrate from the isthmus to the ampulla during the progression of fertilization^61^,^62^,^63^. Thus, the capacitated spermatozoa show attraction by exposing the functional receptor NPR2, and travel from the isthmus of the oviduct to the ampulla during ovulation.

Chemoattractant binding results in a transient cGMP-induced influx of extracellular Ca^2+^ via Ca^2+^ channels in marine invertebrates^4^,^5^,^23^,^64^. It plays a crucial role in the regulation of sperm attraction^65^,^66^,^67^. Cyclic GMP-sensitive CNG channels have been detected in mammalian sperm^68^,^69^, which control the Ca^2+^ entry into spermatozoa^41^,^69^. In the present study, NPPC increased cGMP levels, and induced Ca^2+^ influx via CNG channels, suggesting that NPPC-elevated Ca^2+^ plays an important role in sperm attraction. The elevation of Ca^2+^ also increases the swimming speed of spermatozoa along the chemoattractant gradient^11^,^18^,^23^. NPPC-induced attraction is accompanied by enhanced sperm motility^27^. Spermatozoa swim along the gradient of NPPC with enhanced speed, which is required for movement along the long oviduct to the oocytes. It is suggested that hyperactivation enables spermatozoa to reach the oocyte by assisting escape from the oviductal sperm reservoir^43^,^70^. CatSper-null mutant spermatozoa that cannot undergo hyperactivation are not able to migrate to the site of fertilization^70^. In the present study, very few *Npr2* mutant sperm arrived at the ampulla after 4 h post mating. Although *Npr2* mutant had no effect on sperm motility, it needs further study whether there is a problem in the percentage of hyperactivated spermatozoa in *Npr2* mutant mice.

**Figure 6:**
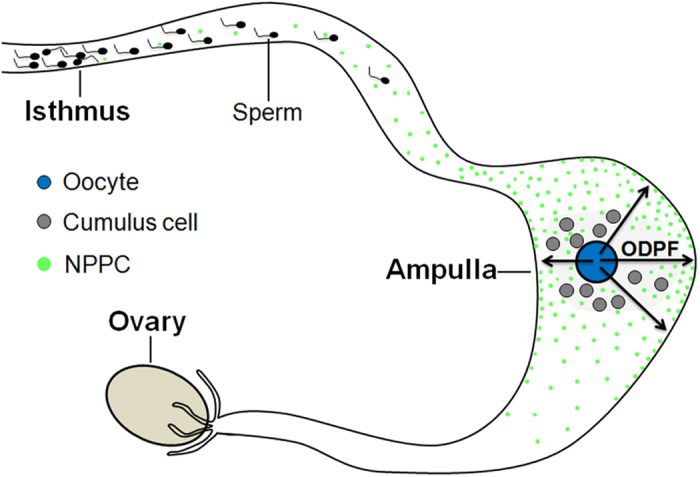
Model depicting the regulation of NPPC by oocyte-derived paracrine factors (ODPF) for sperm attraction in oviduct. See text for details.

This study demonstrates a complex regulatory network involving oocytes, spermatozoa and the oviductal epithelium in ampulla (Fig. 6). The oviductal epithelium in ampulla secretes NPPC that provides a stable and persistent gradient of attraction in the oviduct. Spermatozoa are attracted to oocytes in response to NPPC. Oocytes promote NPPC expression, which is critical for fertilization. Our findings may have potential clinical implications for the treatment of infertility and for contraception.

## Materials and Methods

### Animals

ICR (CD1) female (8 weeks old) and male (3–6 months old) mice were purchased from the Laboratory Animal Center of the Institute of Genetics and Developmental Biology (Beijing, China). Mice were housed under controlled temperature (23 ± 2 °C) and light (12 h light/12 h darkness) with food and water *ad libitum* in an air-conditioned room. Ovulation was induced by treating the diestrus female mice with 5 IU equine chorionic gonadotropin (eCG), followed by 5 IU of human chorionic gonadotropin (hCG) 46 h to 48 h later. CBACa A^W-J^/A-*Npr2*^cn-2J^/GrsrJ (*Npr2*^cn-2J^/*Npr2*^cn-2J^) mice were produced by crossing heterozygous males and females obtained from The Jackson Laboratory (Bar Harbor, ME, USA). *Npr2*^cn-2J^ is a loss-of-function mutation at the *Npr2* locus^30^,^71^. The homozygous mutant mice exhibit an achondroplastic phenotype readily distinguishable from wild-type counterparts as early as 6 days of age. Mice were maintained according to the Guide for the Care and Use of Laboratory Animals (Institute for Learning and Animal Research at China Agricultural University). All the methods were approved by the Institutional Animal Care and Use Committee of China Agricultural University.

### Isolation of ampulla, isthmus, and uterotubal junction

The ampulla, isthmus, and uterotubal junction were isolated from the oviducts of mice at diestrus or estrus using a pair of 26 gauge needles under a stereomicroscope as reported previously^14^,^26^. In some experiments, the ampulla, isthmus, and uterotubal junction were isolated from ovulating mice, or from unilaterally ovariectomized mice. The unilaterally ovariectomized mice were used after two weeks of recovery. All the samples were immediately frozen in liquid nitrogen and stored at −80 °C until mRNA expression was analyzed as described below.

### Co-culture of oviductal ampullae with oocyte-cumulus complexes (OCCs) and oocytectomized (OOX) cumulus cells and denuded oocytes

Ampullae were cultured in a medium of bicarbonate-buffered MEMα with Earle’s salts, supplemented with 75 μg/mL penicillin G, 50 μg/mL streptomycin sulfate, 0.23 mM pyruvate, and 3 mg/mL BSA. All the reagents were purchased from Sigma-Aldrich (St. Louis, MO, USA), unless otherwise stated. The ampullae were isolated from pre-ovulatory mice (at 11 h post hCG), and OCCs were collected from the ampullae of ovulating mice (at 13 h post hCG). Groups of four ampullae with or without 100 OCCs were transferred into 50 μL drops. In some experiments, denuded oocytes (DOs) were obtained by removing the cumulus cells with 1 mg/mL hyaluronidase. OOX cumulus cells were produced by microsurgically removing oocytes, but not the zona pellucida, from the OCCs. OOX cumulus cells or oocytes obtained from 150 OCCs were co-cultured with four ampullae in a 50 μl drop. Occasionally, the ampullae were cultured with a recombinant human growth differentiation factor 9 from *E. coli* (GDF9, 500 ng/mL), recombinant human bone morphogenetic protein 15 from CHO cells (BMP15, R&D Systems, Minneapolis, MN, USA. 500 ng/mL) and/or recombinant human fibroblast growth factor 8B from HEK 293 cells (FGF8, 100 ng/mL). All the cultures were incubated at 37 °C under 5% CO_2_. Each experiment was repeated at least three times. At the end of 3 h, the ampullae were frozen immediately in liquid nitrogen and stored at −80 °C until analysis of mRNA expression as described below.

### Quantitative RT-PCR

Total RNA was isolated and purified using the RNeasy micro-RNA isolation kit (Qiagen, Valencia, CA, USA) according to the manufacturer’s instructions. Reverse transcription was performed directly after RNA isolation using the QuantiTect reverse transcription system (Qiagen). Real-time PCR was conducted to quantify steady-state mRNA levels using an ABI 7500 real-time PCR instrument (Applied Biosystems, Foster City, CA, USA). The levels of mRNAs were first normalized to the expression levels of a housekeeping gene, ribosomal protein L19 (*Rpl19*), and expressed relative to a control group whose expression level was set at 1. PCR primers for *Nppc, Npr2* and *Rpl19* were reported previously^30^,^72^. *Nppa* and *Nppb* primer sequences were indicated in Supplementary Table S2. The specificity of the primers was confirmed by sequencing of the band after electrophoresis.

### Measurement of NPPC levels

Samples were prepared using a modified method reported previously^56^. The ampulla, isthmus, and uterotubal junction were separated from ovulating mice (at 13 h post hCG). Each segment from 10 mice in a single experiment was transferred into a 1.5 mL centrifuge tube and stored at −80 °C. Prior to protein analysis, the tissues in 100 μL of 1.0 M acetic acid were boiled for 5 min, and homogenized on ice using a tissue homogenizer (T10 basic, IKA, Germany) and lysed with ultrasonic cell disruptor (Scientz-IID, Ningbo, China). We added 500 μL of MeOH to solubilize the lipids. The samples were centrifuged at 20,000× *g* at 4 °C for 30 min, and the supernatant contained 1.0–5.0 mg of protein were frozen. For each sample, 100, 50 and 10 μg of the protein extracts were lyophilized and assayed by fluorescent enzyme immunoassay kits (Phoenix Pharmaceuticals, Belmont, CA, USA) according to the manufacturer’s instructions.

### *In situ* hybridization

The ampullae were isolated from ovulation-induced mice, embedded rapidly in optimal cutting temperature compound (OCT; Sakura, Tokyo, Japan), and frozen at −80 °C prior to analysis. Frozen sections (10 μm) were prepared using a CM 1950 cryostat microtome (Leica, Wetzlar, Germany) and mounted on Super-Frost Plus slides (Thermo Scientific, Waltham, MA, USA) for *in situ* hybridization using DIG-labeled riboprobes as reported previously^73^. Briefly, sections were hybridized with DIG-cRNAs (1.5 μg/mL) overnight after pre-hybridization, and incubated with anti-digoxigenin-AP. The sections were incubated with NBT/BCIP (nitroblue tetrazolium chloride/5-bromo-4-chloro-3-indolyl-phosphate, toluidine salt; Roche Diagnostics, IN, USA) to detect bound digoxigenin-AP. Sequences of PCR primers used to amplify cDNA templates for preparation of *in situ* probes were reported previously^30^.

### Sperm preparation

Sperm collection and incubation were carried out in Tyrode (T6) medium comprising: 114.0 mM NaCl, 3.2 mM KCl, 0.5 mM MgCl_2_, 2.0 mM CaCl_2_, 0.4 mM NaH_2_PO_4_, 24.9 mM NaHCO_3_, 5.6 mM glucose, 0.5 mM sodium pyruvate, 10 mM sodium DL-lactate (60% wt/vol), 10 mM hepes, 0.01 mg/mL phenol red and 10 mg/mL bovine serum albumin (BSA). A T6 medium without BSA was used for the measurement of Ca^2+^ levels. Caudal epididymal sperm cells were obtained from male mice. Excised caudal epididymis was rinsed and fragmented in a 400 μL drop of T6 medium. Immediately following dispersion of spermatozoa into the medium, the whole sperm suspension was transferred to a 1.5 mL plastic tube. For *in vitro* capacitation, the spermatozoa were incubated for 1.5 h in T6 medium in a 37 °C incubator under 5% CO_2_.

### Identification and localization of NPPC receptor

The NPPC receptor sites on spermatozoa were identified by FAM-labeled (the Mono-5-(and 6)-carboxyfluorescein label) NPPC (FAM-NPPC, Phoenix Pharmaceuticals, Belmont, CA, USA). Fresh spermatozoa and capacitated or chemoattractant-treated spermatozoa were incubated with 100 nM FAM-NPPC for 30 min to ensure full reaction. The spermatozoa were fixed with 2% paraformaldehyde for 5 min, and washed three times with PBS, 5 min each time at 300× *g* to remove unbound ligand. Fluorescent-labeled ligand binding was assessed using a confocal laser-scanning microscope (Nikon A1R, Tokyo, Japan). Cells with uneven dye loading were excluded from the analysis. Competition experiments were performed by incubating the spermatozoa for 30 min in the presence of 100 nM FAM-NPPC and 1 μM of unlabeled NPPC.

### Sperm motility

Capacitated spermatozoa were incubated in T6 medium without (control) or with 1 nM NPPC for 30 min. The sperm motion was determined using a CASA system (Version.12 CEROS, Hamilton Thorne Research, Beverly, MA, USA). The parameters included curvilinear velocity (VCL), straight-line velocity (VSL), average path velocity (VAP), amplitude of lateral head displacement (ALH), beat cross frequency (BCF), percentage of linearity (LIN; VSL/VCL × 100%), and percentage of straightness (STR; VSL/VAP × 100%). Progressive motility (% of motile spermatozoa with average path velocity (VAP) ≥ 50 μm/s, and straightness ratio (STR) ≥ 80%) were determined. Cells were detected with a minimal contrast of 50 and minimal cell size of 4 pixels. Sixty frames were acquired at a frequency of 60 Hz. Almost 10 random fields were evaluated for each sample, accounting for a total of 500 spermatozoa.

### Western blot

A total of 2 × 10^8^ spermatozoa were collected from the caudal epididymis of adult male mice as described above. The sperm heads and tails were separated according to the previous study^2^. Briefly, the spermatozoa were snapped frozen in liquid nitrogen and diluted with 600 μL of PBS. The sperm suspension was passed thirty times through a 26-gauge needle on ice and centrifuged at 1000× *g* for 5 min at 4 °C to precipitate the sperm heads. Sperm flagella were recovered from the supernatant by centrifugation at 10,000× *g* for 20 min at 4 °C. NPR2 was immunoprecipitated as reported previously^36^. Briefly, the samples were diluted to 600 μL in an immunoprecipitation buffer containing 50 mM Tris-HCl (pH 7.5), 100 mM NaCl, 50 mM NaF, 10 mM NaH_2_PO_4_, 2 mM EDTA, 1% NP-40, 0.1% SDS, 0.5% sodium deoxycholate, 1 μM microcystin-LR, and protease inhibitors. The samples were incubated with 0.6 μL of rabbit polyclonal NPR2 antiserum provided by Lincoln R. Potter^36^ for 1 h at 4 °C, and then with 25 μl of washed protein AG magnetic beads (Thermo Fisher Scientific, Rockford, IL, USA) overnight at 4 °C. The beads were washed with Tris-buffered saline with Tween 20 (TBST) and resuspended in Laemmli sample buffer. The proteins were eluted by heating at 100 °C for 10 min.

The immunoprecipitated samples from sperm heads and flagella were separated by SDS-PAGE with 5% (w/v) stacking gel for 40 min at 60 V and 6% (w/v) separating gel for 90 min at 100 V, and electrically transferred to a PVDF membrane. The membranes were blocked with TBS containing 0.1% Tween-20 and 2% milk. Blots for NPR2 were probed with a 1:10,000 dilution of NPR2 antiserum overnight at 4 °C. After washing, the membranes were incubated with 1:500 dilution of Clean-Bolt IP Detection Reagent coupled to HRP (Thermo Fisher Scientific, Rockford, IL, USA). The blots were visualized using Tanon 5200 chemiluminescent imaging system (Tanon, Shanghai, China). The controls included protein samples from sperm heads and flagella separated by SDS-PAGE. Blots were probed with polyclonal anti-rabbit β-actin antibody (1:1000; Cell Signaling Technologies, Danvers, MA, USA) and incubated with goat anti-rabbit IgG (1:5,000; Pierce Biotechnology Inc., Rockford, IL, USA) in TBST for 60 min at room temperature.

### Assay of sperm accumulation in the capillary

The method for sperm accumulation analysis was modified from previous studies using a series of polyethylene tubes measuring 3 cm in length and 1 mm in diameter, and Petri dishes with a diameter of 9 cm^25^,^26^. Briefly, a 150 μL droplet of capacitated spermatozoa at a concentration of 1–2 × 10^6^ cells/mL was placed in the middle of the Petri dishes. The capillaries with one side sealed were filled with T6 medium supplemented with or without various concentrations of NPPC (0.01–10 nM), and the open side was connected to the droplet. The capillaries and the droplet were covered by mineral oil. A droplet containing 0.1 nM NPPC was used for descending gradient (DG) analysis. After incubation for 20 min, the spermatozoa in the capillaries were counted by a hemocytometer under the microscope.

### Measurement of cGMP levels

After capacitation, spermatozoa suspended in 50 μL (5 × 10^6^ cells/ml) were incubated with or without 1 nM NPPC for 20 min. They were collected and solubilized in 100 μL of 0.1 M HCl on ice for at least 10 min. These samples were snap frozen in liquid nitrogen, transferred and stored at −80 °C. For cGMP assay, the samples were thawed and centrifuged at 12,000× *g* for 5 min, and the supernatant was collected in a tube and dried in an oven at 60 °C. The levels of cGMP were determined using the protocol described in the previous study^30^ using cGMP-enzyme immunoassay kits obtained from Cayman Chemicals (Ann Arbor, MI, USA).

### Imaging analysis of the intracellular Ca^2+^ levels

Ca^2+^ levels were detected by a method modified from the previous study^41^. Capacitated spermatozoa were loaded with 5 μM Fluo 3-AM (Dojindo Laboratories, Kumamoto, Japan) and 0.06% pluronic F-127 for 30 min at 37 °C in the dark. The sperm suspension (3–5 × 10^5^ cells/mL) was placed on glass coverslips treated with polylysine at 0.01% in the recording chamber and after 5 min the external solution was infused to wash the supernatant. Imaging analysis of Ca^2+^ response in motile spermatozoa was monitored using the confocal laser-scanning microscope (Nikon, A1R). We used a sample frequency of 1 Hz, and recorded for 3–4 min after stimulus. The elevation of Ca^2+^ in the sperm head and flagellar midpiece was expressed as *F*_max_/*F*_0_ ratios after background subtraction, where *F*_max_ represented the maximum peak of florescence signal intensity, and *F*_0_ denoted the baseline, calculated as the average of the first 15 sec prior to stimulus application. All Ca^2+^ imaging experiments were carried out at 37 °C. Cells with uneven dye loading were excluded from the analysis. NPPC was used at 0.1 nM, and the inhibitor *l-cis-*Diltiazem was used at 50 μM with a pre-incubation of 15 sec. NPPC and the inhibitor were dropped into the recording chamber using pipette tips and the recordings were conducted in the continuous presence of stimuli. Ca^2+^-free experiments were conducted using Ca^2+^-free T6 medium without BSA, obtained by omitting Ca^2+^ and adding 1 mM EGTA.

### Artificial insemination

Eight-week-old female mice were induced to ovulate using a slight modification of the procedure described above. The doses of both eCG and hCG were 2 IU. Artificial insemination was conducted at 13 h post hCG by depositing capacitated spermatozoa of *Npr2*^wt^/*Npr2*^cn-2J^ heterozygote or *Npr2*^cn-2J^/*Npr2*^cn-2J^ mutant male mice (approximately 3 months old) into the female uterus via cervix. The volume of spermatozoa inseminated per female was 50 μL containing 3–5 × 10^6^ spermatozoa. Subsequently, the female mice were mated with vasoligated male immediately to develop a vaginal plug. In some experiments, capacitated spermatozoa from CD1 males were pre-incubated with 50 μM *l-cis*-D for 10 min before artificial insemination. After 4 h, the ampulla was separated, and the spermatozoa and OCCs were flushed out of the ampulla. The oocytes and cumulus cells were diffused with 1 mg/ml hyaluronidase. The spermatozoa in the medium were counted. 36 h later, embryos and presumptive zygotes in the oviduct were collected and captured on a 20× objective. The two-cell embryo rate was calculated.

### Statistical analysis

All the experiments were repeated a minimum of three times. Results were expressed as mean ± SEM. Significant differences between experimental and control groups were analyzed using a Student’s *t*-test. A P value of less than 0.05 was considered statistically significant.

## Additional Information

**How to cite this article**: Kong, N. *et al*. Natriuretic peptide type C induces sperm attraction for fertilization in mouse. *Sci. Rep.* **7**, 39711; doi: 10.1038/srep39711 (2017).

**Publisher's note:** Springer Nature remains neutral with regard to jurisdictional claims in published maps and institutional affiliations.

## Supplementary Material

Supplementary Information

## Figures and Tables

**Table 1:** Effect of NPPC on sperm motility.

Treatment	Control	NPPC
Progressive motility (%)	20.6 ± 0.9	34.7 ± 0.9*
VAP (μm/s)	110.4 ± 1.9	130.1 ± 1.2*
VSL (μm/s)	73.9 ± 1.3	93.1 ± 1.4*
VCL (μm/s)	216.8 ± 3.9	221.2 ± 1.9
ALH (μm)	9.2 ± 0.2	9.0 ± 0.2
BCF (HZ)	24.4 ± 0.5	23.6 ± 0.4
STR (%)	61.0 ± 0.8	69.6 ± 0.5
LIN (%)	34.3 ± 0.6	44.0 ± 1.0

VAP, average path velocity; VSL, straight line velocity; VCL, curvilinear velocity; ALH, amplitude of lateral head displacement; BCF, beat cross frequency; STR, percentage of straightness; LIN, percentage of linearity; progressive motility (% of motile spermatozoa with VAP ≥ 50 μm/s and STR ≥ 80%). Bars indicate the mean ± SEM of three experiments; *n* = 500 for each sample. ^*^P < 0.05.
