# Supplementary material for: Natriuretic peptide type C induces sperm attraction for fertilization in mouse
Source: Sci Rep. 2017 Jan 5;7:39711. doi: 10.1038/srep39711 (PMC5214687; doi:10.1038/srep39711)

Supporting Information

Natriuretic peptide type C induces sperm attraction for fertilization in mouse

Nana Kong[1], Xiaoting Xu[1], Yu Zhang[1], Yakun Wang[1], Xiaoqiong Hao[1], Yu Zhao[1], Jie Qiao[2], Guoliang Xia[1] and Meijia Zhang[1,*]

[1]State Key Laboratory for Agrobiotechnology, College of Biological Sciences, China Agricultural University, Beijing 100193, China. [2]Department of Obstetrics and Gynecology, Reproductive Medical Center, Peking University Third Hospital, Beijing 100191, China.

N.K. and X.X. contributed equally to this work.

Correspondence: Meijia Zhang, State Key Laboratory for Agrobiotechnology, College of Biological Science, China Agricultural University, Beijing 100193, China. Tel: 86 10 6273 2694. E-mail: zmeijia@cau.edu.cn.

Table S1. Compare of sperm motility in NPR2 heterozygote and mutant mice

Treatment	NPR2 heterozygote	NPR2 mutant
Progressive (%)	21.3 ± 1.3	19.4 ± 0.7
VAP (μm/s)	113.1 ± 2.4	116.2 ± 4.2
VSL (μm/s)	74.2 ± 2.1	76.6 ± 4.9
VCL (μm/s)	214 ± 6.6	223.3 ± 5.5
ALH (μm)	8.9 ± 0.4	8.5 ± 0.2
BCF (HZ)	25.2 ± 0.9	23.9 ± 0.6
STR (%)	61.8 ± 1.4	63.9 ± 0.6
LIN (%)	35.2 ± 1.2	36.4 ± 0.5

VAP, average path velocity; VSL, straight line velocity; VCL, curvilinear velocity; ALH, amplitude of lateral head displacement; BCF, beat cross frequency; STR, percentage of straightness; LIN, percentage of linearity; progressive motility (% of motile spermatozoa with VAP ≥ 50 μm/s and STR ≥ 80%). Bars indicate the mean ± SEM of three experiments; n = 500 for each sample.

Table S2. Sequence of PCR primers used for qRT-PCR

Primer	Forward	Reverse
Nppa	CCAGGCCATATTGGAGCAAAT	TTCTCCTCCAGGTGGTCTAGCA
Nppb	AGCTGCTTTGGGCACAAGAT	CAGGCAGAGTCAGAAACTGGAGTCT

Fig. S1. Comparison of amino sequences of mouse natriuretic peptides (NPs) and the confirmed chemoattractants. Amino acid sequence analysis revealed that mouse natriuretic peptide type A (NPPA), type B (NPPB) and type C (NPPC) exhibited similar characters to the chemoattractant peptides resact and asterosap in marine invertebrates: there are glycine-rich peptides (red), circular by the formation of an intramolecular disulfide bond between cysteines (green), and binding to a receptor-type guanylyl cyclase (GC) to stimulate the synthesis of cGMP.

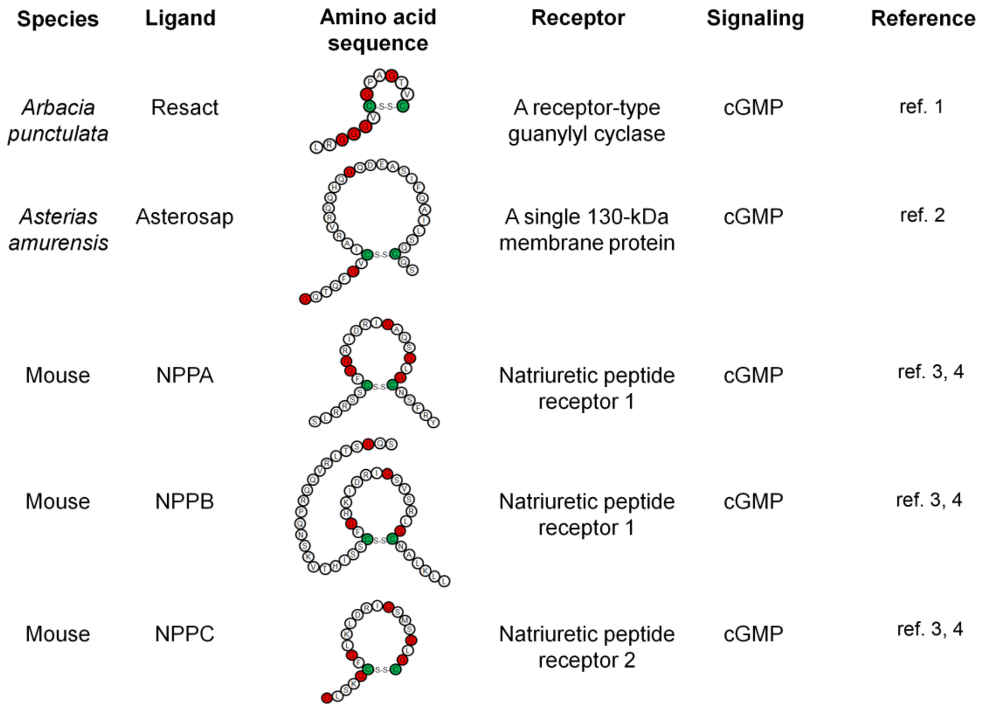

References

1 Kaupp, U. B. *et al*. The signal flow and motor response controling chemotaxis of sea urchin sperm. *Nat Cell Biol* **5**, 109-117 (2003).

2 Nishigaki, T., Chiba, K. & Hoshi, M. A 130-kDa membrane protein of sperm flagella is the receptor for asterosaps, sperm-activating peptides of starfish Asterias amurensis. *Dev Biol* **219**, 154-162 (2000).

3 Potter, L. R., Abbey-Hosch, S. & Dickey, D. M. Natriuretic peptides, their receptors, and cyclic guanosine monophosphate-dependent signaling functions. *Endocr. Rev.* **27**, 47-72 (2006).

4 Zhang, Y. *et al*. Porcine natriuretic peptide type B (pNPPB) maintains mouse oocyte meiotic arrest via natriuretic peptide receptor 2 (NPR2) in cumulus cells. *Mol Reprod Dev* **81**, 462-469 (2014).

Fig. S2. *Nppc* mRNA is expressed predominantly in the ampulla of estrous mice. (**a**) Comparison of natriuretic peptides mRNA levels in the uterotubal junction (Junction), isthmus and ampulla from the oviducts of estrous mice. The mean value in ampulla group was set as 1. Bars indicate mean ± SEM of four experiments. $*P < 0.05$; $**P < 0.01$. (**b**) Comparison of *Nppa*, *Nppb* and *Nppc* mRNA levels in the ampulla collected from estrous mice. Bars indicate mean ± SEM of four experiments. $**P < 0.01$.

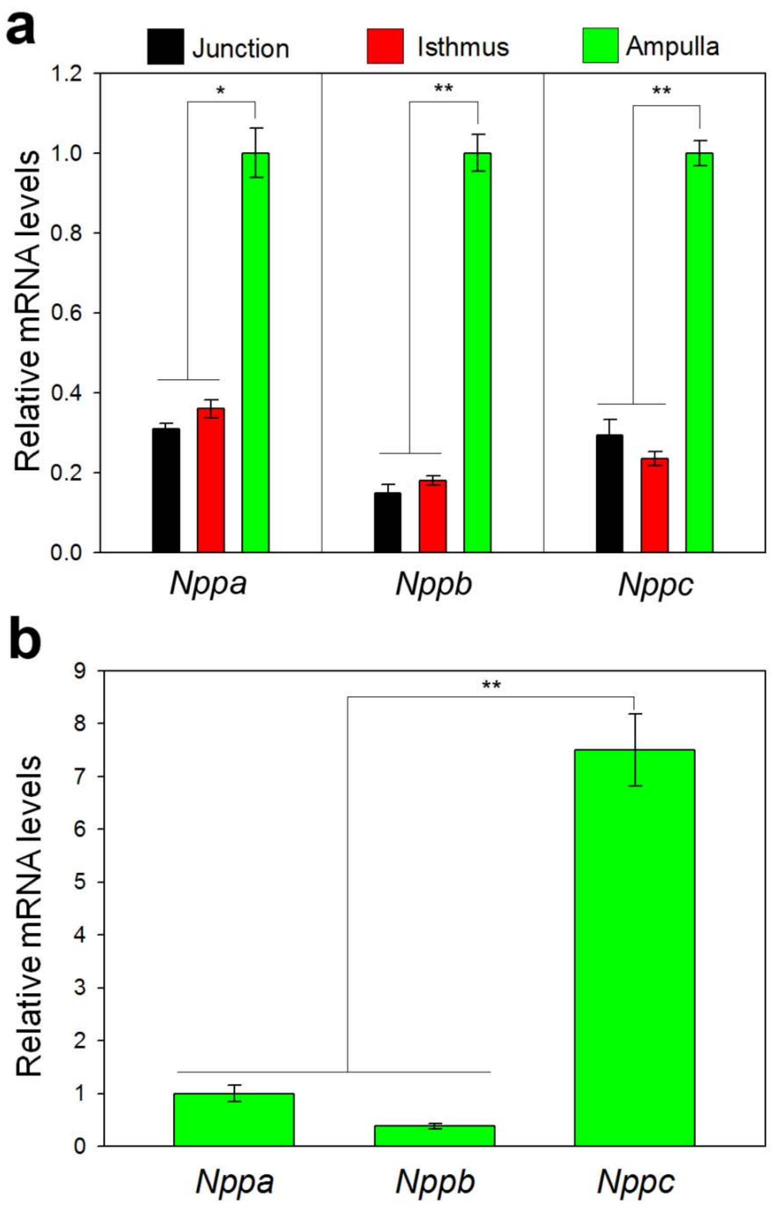

Fig. S3. NPR2 is expressed in spermatozoon. (**a**) Comparison of *Nppc* and *Npr2* mRNA levels in fresh spermatozoa. Bars indicate mean ± SEM of three experiments. ***$P < 0.001$. (**b**) Comparison of *Npr2* mRNA levels in fresh and capacitated spermatozoa. No obvious change was indicated. (**c-e**) Capacitated spermatozoa were incubated with 100 nM FAM-NPPC for 30 min, and the fluorescent ligand binding was assessed by a confocal laser-scanning microscope. Representative images showed fluorescence (**c**), bright (**d**) and merge (**e**) fields. Scale bars, 20 µm. (**f**) Comparison of FAM-NPPC binding in fresh, capacitation and attraction spermatozoa. Bars indicate the mean ± SEM of three experiments. $n = 500$ for each group. **$P < 0.01$; ***$P < 0.001$.

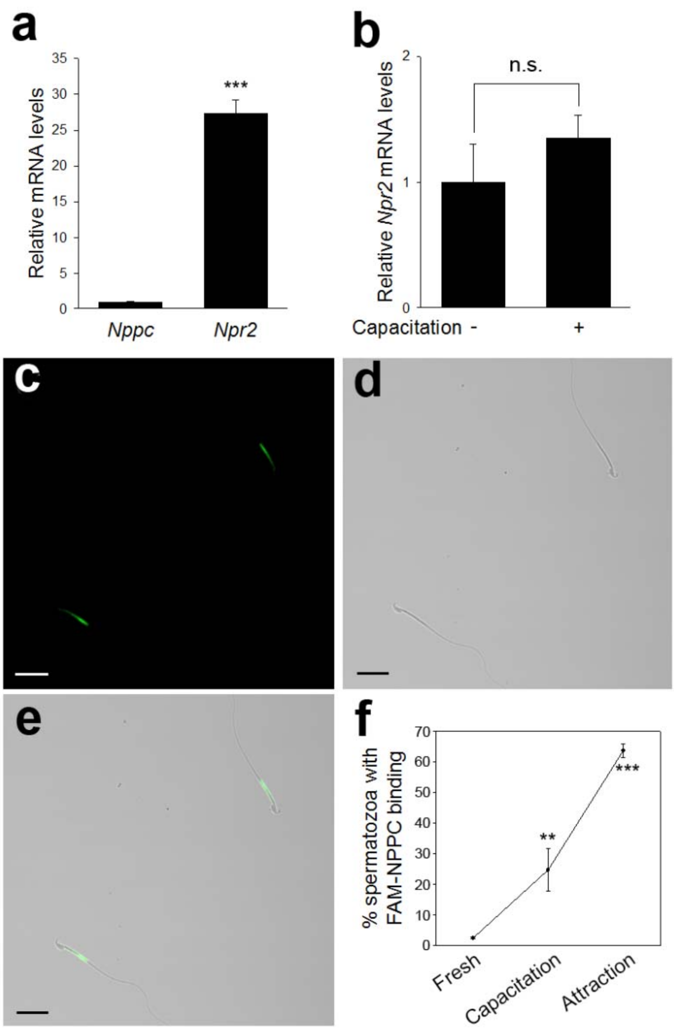

Fig. S4. Failure in FAM-NPPC binding to spermatozoa from *Npr2* mutant mice. Capacitated spermatozoa from *Npr2* heterozygote and mutant mice were incubated with 100 nM FAM-NPPC for 30 min, and the fluorescent ligand binding was assessed by a confocal laser-scanning microscope. (**a**-**c**) Representative images showed fluorescence (**a**), bright (**b**) and merge (**c**) fields. Scale bars, 20 μm. (**d**) Comparison of FAM-NPPC binding in capacitated spermatozoa from *Npr2* heterozygote and mutant mice. Bars indicate the mean ± SEM of three experiments. n = 500 for each group. **$P < 0.01$.

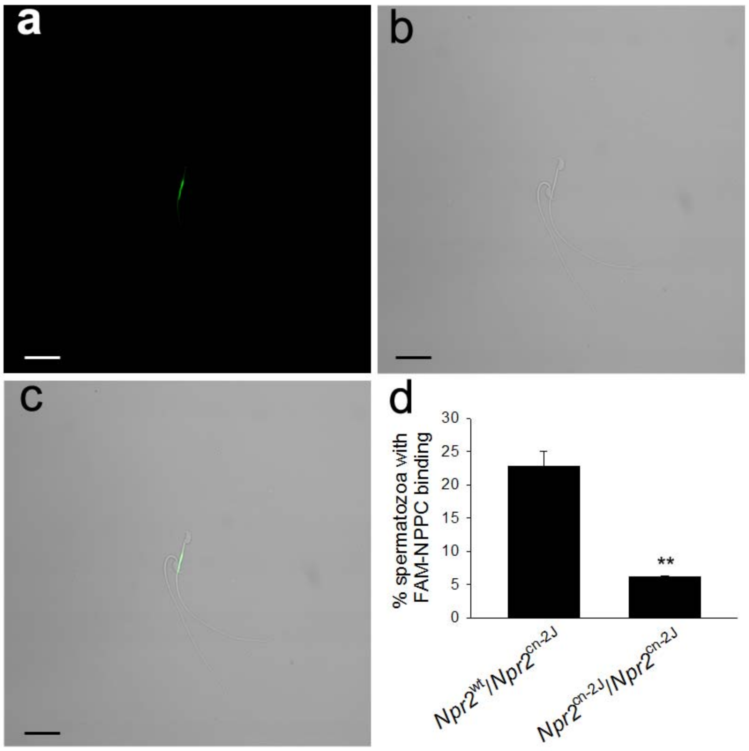

Fig. S5. Time lag in the Ca^{2+} influx induced by NPPC between flagellar midpiece and head of mouse spermatozoon. Time measurement analysis showing the propagation of elevated Ca^{2+} from the midpiece of flagellum to the head after application of 0.1 nM NPPC. The zero time was defined as the time of NPPC addition. Representative images are presented in a pseudocolor format. Scale bars, 20 μm.

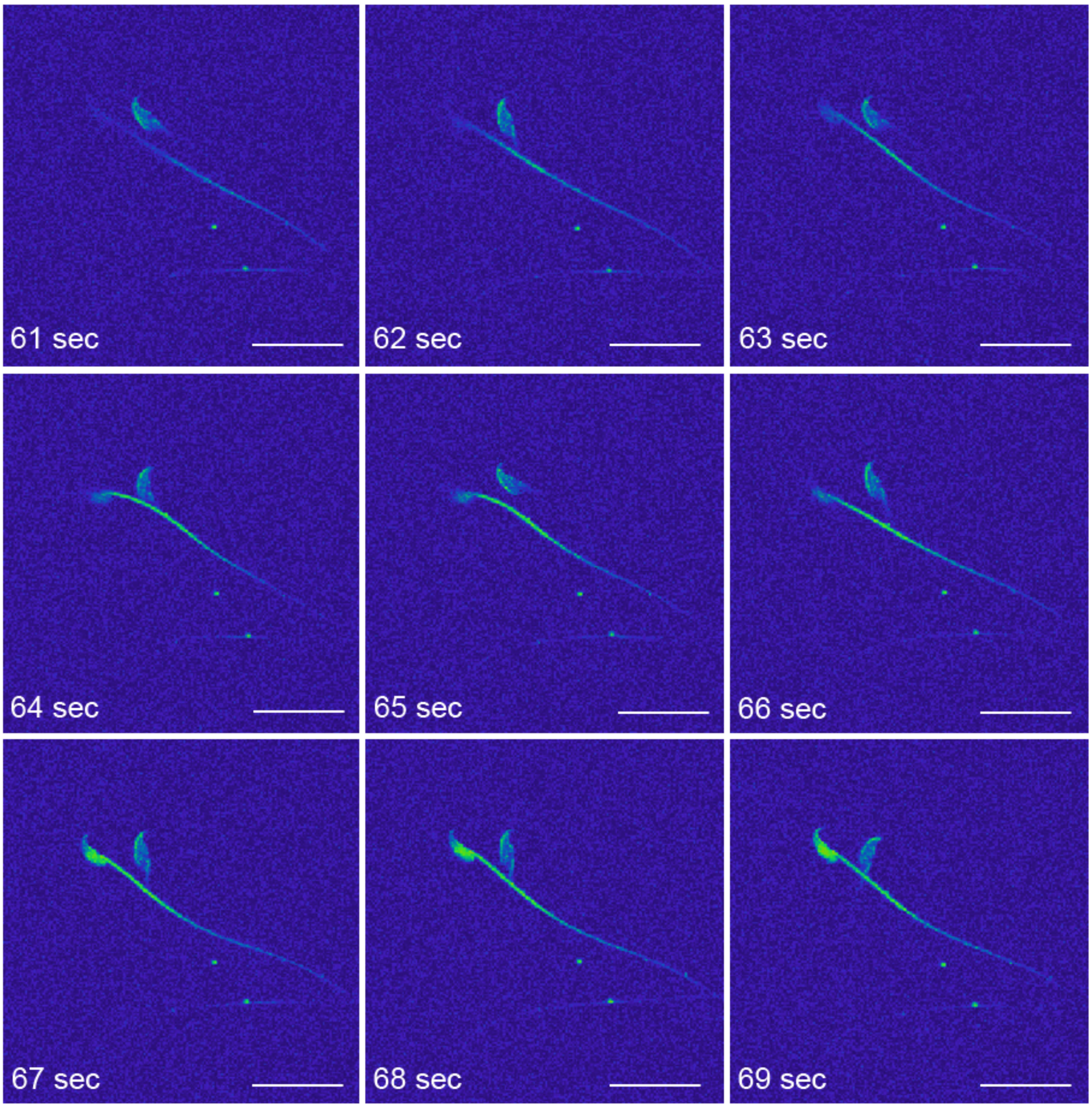

Fig. S6. *l-cis*-Diltiazem blocked NPPC-induced sperm accumulation. (**a**) Sperm accumulation in the capillaries in the groups of control, NPPC (0.1 nM), *l-cis*-Diltiazem (*l-cis*-D, 50 μM) and NPPC + *l-cis*-Diltiazem. Bars indicate the mean ± SEM of three experiments. (**b**) Sperm accumulation in the oviductal ampullae. Bars indicate the mean ± SEM of four experiments. *** $P < 0.001$.

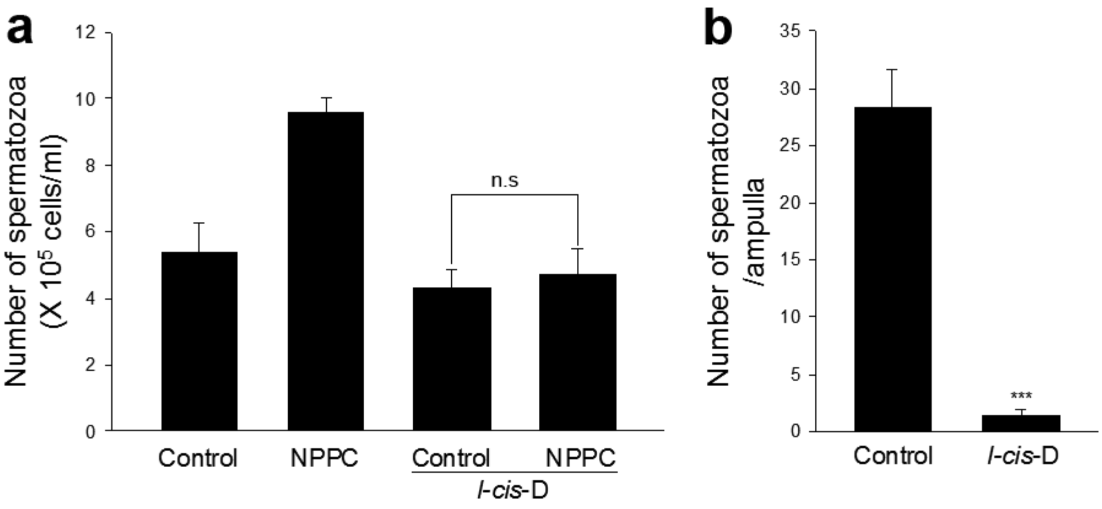

Supplement: Supplementary Information [file srep39711-s1.pdf]
